# Microgelation imparts emulsifying ability to surface-inactive polysaccharides—bottom-up vs top-down approaches

**DOI:** 10.1038/s41538-018-0023-7

**Published:** 2018-08-20

**Authors:** Toya Ishii, Kentaro Matsumiya, Mai Aoshima, Yasuki Matsumura

**Affiliations:** Laboratory of Quality Analysis and Assessment, Division of Agronomy and Horticultural Science, Graduate School of Agriculture, Kyoto University, Gokasho, Uji, Kyoto, 611-0011 Japan

**Keywords:** Colloids, Biosurfaces, Biopolymers, Gels and hydrogels

## Abstract

In order to impart emulsifying ability to gel-forming polysaccharides that have not been used as emulsifying agents, three kinds of polysaccharides, agar, curdlan, and gellan gum were converted to microgels by different gelation methods via the bottom-up and top-down approaches. We clearly demonstrated that agar and curdlan acquired the ability to emulsify an edible oil by microgel formation. Among the colloidal properties of microgel suspensions such as microstructure, particle size, zeta-potential, viscosity, and surface hydrophobicity, we pointed out the importance of particle size on the emulsifying ability of polysaccharide-based microgels. The creaming behavior of the microgel-stabilized emulsions depended on the polysaccharide types and microgel preparation methods. The emulsion stability against oil droplet coalescence was extremely high for agar and curdlan microgel-stabilized emulsions during storage in the static condition, whereas different stability was observed for both the emulsions, that is, the curdlan microgel-based ones were more resistant to dynamic forcible destabilization by centrifugation than the agar ones, which can be attributed to the surface hydrophobicity of the microgels.

## Food Emulsion: Microgelation turns polysaccharides into emulsifying agents

Polysaccharides are widely recognized as gelling agents while rarely used alone as emulsifying agents due to their surface inactiveness. Now Kentaro Matsumiya and coworkers from Kyoto University report the microgelated polysaccharide particles can emulsify soybean oil, forming so-called microgel-based Mickering emulsions. It was found that large microgels formed by agar and curdlan exhibited better emulsifying abilities compared to small gellan gum microgels, regardless of the preparation methods. The creaming behaviors varied with the changing polysaccharide types and microgel preparation methods. The static stability was quite high for all emulsions while their dynamic stability was different, which can be attributed to the surface hydrophobicity difference. It is noteworthy that the microgelation turned surface-inactive polysaccharides into effective emulsifying agents. The low-allergenic polysaccharide food emulsions might find usage practically soon.

## Introduction

Many food products such as dressings, coffee creams, and white sauces exist as an oil-in-water (O/W) emulsion where small oil droplets are dispersed in an aqueous continuous phase.^1^ These oil droplets are mainly stabilized by surface-active biopolymers like milk and egg yolk proteins due to their high emulsifying ability occasionally together with small-molecule surfactants for food production,^2,3^ whilst such animal proteins often require much higher energy input to be produced than plant resources; for example, the required fossil fuel for 1 kcal of the animal and plant protein resources are 25 and 2.2 kcal, respectively.^4^ In addition to this, the major origins of food proteins, e.g., milk, egg, wheat, and soybean, are widely recognized as allergens that need special attentions according to the Codex Committee on Food Labelling.^5,6^ To avoid the allergic reactions, a lot of low-allergenicity food products such as gluten-free breads and noodles have been extensively developed especially for wheat flower products, often combined with polysaccharide-based food hydrocolloids to improve their textual quality.^7–10^ Such low-allergenic polysaccharides originated from plants and microorganisms are eco-friendly as well, but they are not applied enough for food emulsions as an effective emulsifying agent.

Food emulsions are thermodynamically unstable systems and tend to destabilize via physical processes such as creaming, flocculation, and coalescence during transportation and in their shelf life. The emulsifying agents applied to formation of the system are at the same time required to delay progress of these phenomena once oil and water are emulsified. Whereas small-molecule emulsifiers adsorb to the oil–water interface to efficiently stabilize the system to oil droplet coalescence throughout the Gibbs–Marangoni effects,^1,11^ macromolecules like proteins form elastic layers on the oil droplet surfaces during emulsification processes to provide the effective steric and electrostatic effects responsible for stability against coalescence.^1,12^ Despite these favorable properties of proteins to stabilize emulsions, the adsorbed proteins are intrinsically highly susceptible to pH value change, salt addition, and processing such as heat and freeze-thaw treatments,^1,13^ and therefore they are often additionally covered with oppositely charged polysaccharides such as pectin, soluble soybean polysaccharide (SSPS), and carrageenan.^14–17^ Although many kinds of polysaccharides play a major role in stabilizing the interface between oil and water particularly in combination with proteins, they are not usually used for food emulsions as a sole emulsifying agent because typical hydrophilic polysaccharide molecules are surface inactive in principle unless they include protein moieties responsible for emulsifying ability like gum arabic and SSPS for emulsions designed as flavor products.^1,12,18–20^

Historically, polysaccharides have been commonly utilized as a thickening agent for various food products in the food industry, e.g., locust bean gum and xanthan gum are often added into sauces and dressings, where the hydroxyl groups of polysaccharide molecules attractively interact with water molecules via hydrogen bonding to increase friction between the molecules leading to viscous characteristics of the solution. Amongst the polysaccharides, agar, curdlan, gellan gum etc. can act as a gelling agent to form a macrogel under appropriate conditions by intermolecular interactions through association of helical domains and co-formation of helices.^21–23^ In the last decade, polysaccharide-based microgels were developed as a delivering agent of drugs to enclose bioactive compounds for controlled releases.^24,25^ On the other hand, macromolecule-based microgels, not regarding polysaccharides, have been increasingly gaining much attention as a novel class of emulsifying agents to obtain stable emulsion systems. The porous and soft microgel-stabilized emulsions referred to as Mickering emulsions,^26^ a recently proposed scientific technical term, whose properties were found to be different from Pickering ones, were initially prepared with poly(*N*-isopropylacrylamide), a synthesized surface-active macromolecule.^27^ The gel particles adsorbed on the oil droplet surfaces form relatively thick interfacial layers between the oil–water interface to introduce enhanced stability of the emulsions. More recently, edible microgels were successfully fabricated from food-grade materials such as whey and soy proteins.^28–30^ In this context, we conceived of an idea that conversion of ordinary states to insoluble microgels effectively imparts emulsifying ability to less surface-active polysaccharides.

The objective of this work is to study effects of microgelation on the emulsifying ability of polysaccharides that have been rarely applied for stand-alone emulsification of food grade oils. Agar, curdlan, and gellan gum which contain few protein moieties and are widely recognized as gelling agents with different sugar units were chosen as the materials for the preparation of microgels. The fabricated microgel particles suspended in an aqueous phase were characterized by colloidal and rheological analyses and then homogenized with soybean oils to create microgel-based Mickering emulsions. They were subjected to particle size and zeta-potential measurements, and visual and microscopic observations to evaluate emulsifying ability of microgels made from polysaccharides, followed by subsequent emulsion stability tests during several months. Microgels produced via bottom-up or top-down approaches were directly compared to elucidate the impact of fabrication processes on the physical stability of emulsions.

## Results

### Colloidal properties of microgel particles

Colloidal properties of dispersed microgel particles were studied because these properties definitely affect emulsifying ability of particles.^28^ We prepared microgels from three types of polysaccharides via the gel formation under diluted conditions as a building-up method (BU) and the mechanical breakage of macrogels as a breaking-down method (BD-C, BD-80, and BD-240). Microstructure, size, and zeta-potential of the particles and viscosity of the overall suspension systems that significantly influence physical stability of emulsions were analyzed.

**Fig. 1:**
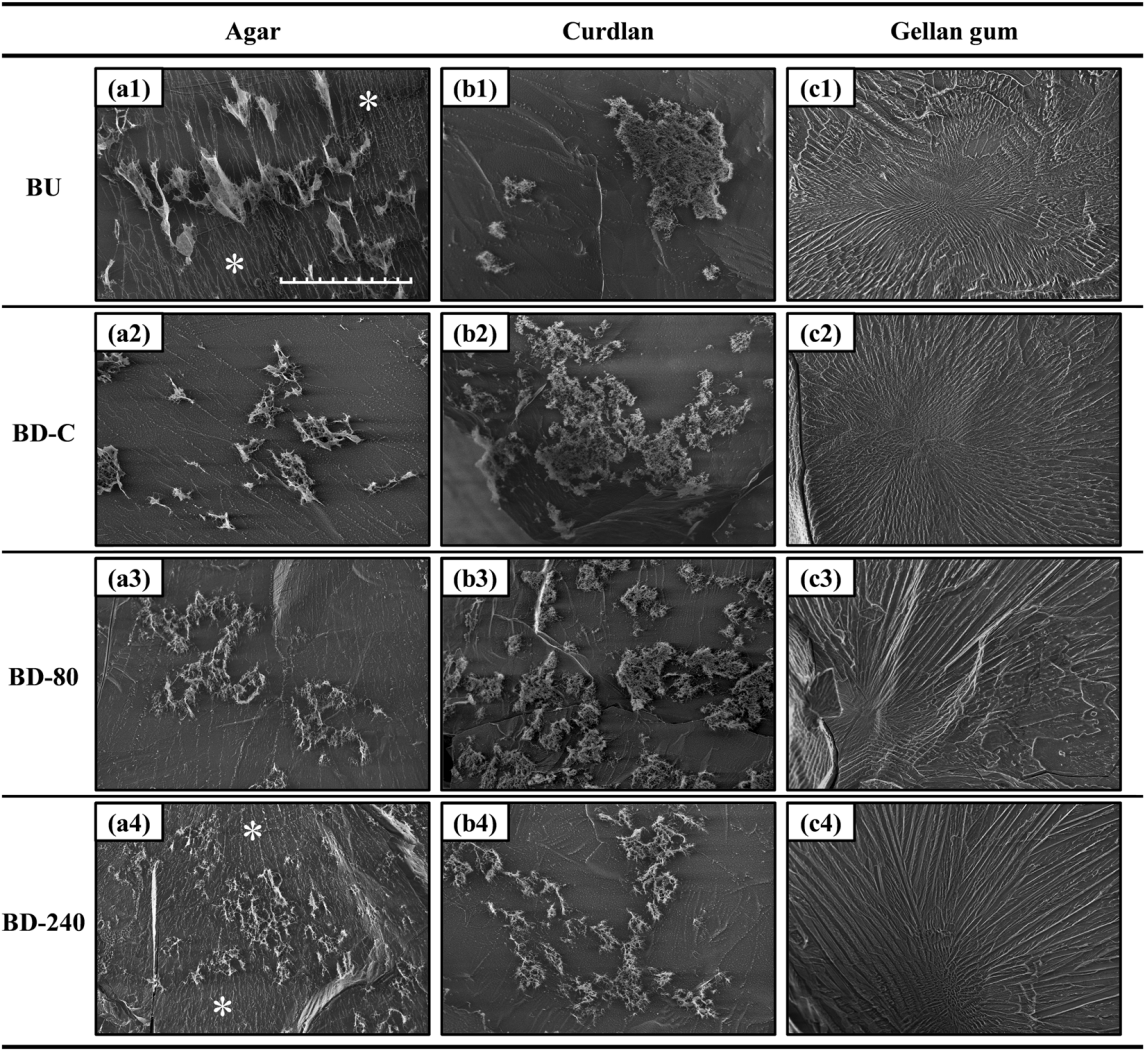
Microstructure of the obtained microgels observed by cryo-SEM. All the sample suspensions (0.125 wt%) filled in a pared metal tube were frozen in a nitrogen slash, sublimated, and subsequently observed at −120 °C. Scale of all images is the same (scale bar = 50 μm)

As all the obtained microgel suspensions were almost transparent and difficult to observe by light microscopy, cryo-SEM was carried out to analyze microstructure of the gel particles. Figure 1 depicts cryo-SEM images of the microgel suspensions captured at a magnification of ×1000. For all the agar microgel suspensions (Fig. 1a1–a4), micro-ordered gel-like particles about 10 μm with remaining networks were found, and BU and BD-240 particles seemed to be surrounded by fibrous materials highlighted by *. For curdlan, BU and BD-C suspensions (Fig. 1b1, b2) included large clusters of several tens of micrometers, whereas BD-80 and BD-240 particles (Fig. 1b3, b4) were mostly smaller compared to such large clusters, meaning that the ultra-high-pressure processing efficiently broke down the curdlan macrogels. These results from the agar and curdlan samples clearly show the formation of microgel particles via both the building-up and breaking-down processes. In the gellan gum suspensions, on the other hand, fibrous networks radially expanded over the observed ice crystal fields, where micro-ordered particles were not found for all the samples prepared (Fig. 1c1–c4). This indicates that gellan gum molecules hardly coagulated even after Ca^2+^ addition under the diluted condition in the BU preparation, while the molecules that once associated by Ca^2+^ addition to form macrogels were atomized into almost soluble submicron states by the breaking-down treatments in BD-C, BD-80, and BD-240 preparations.

In order to evaluate the particle size of the agar, curdlan, and gellan gum microgels, we carried out particle size measurements by DLS and laser-diffraction techniques. However, presumably due to insufficient low scattering light intensity of the transparent polysaccharide microgel particles, any reproducible and reliable values without multiple light scattering were not successfully obtained for almost all the samples. To avoid such a problem, another non-light-dependent method based on Coulter principle with electrolytes was applied for the sample analyses.

**Fig. 2:**
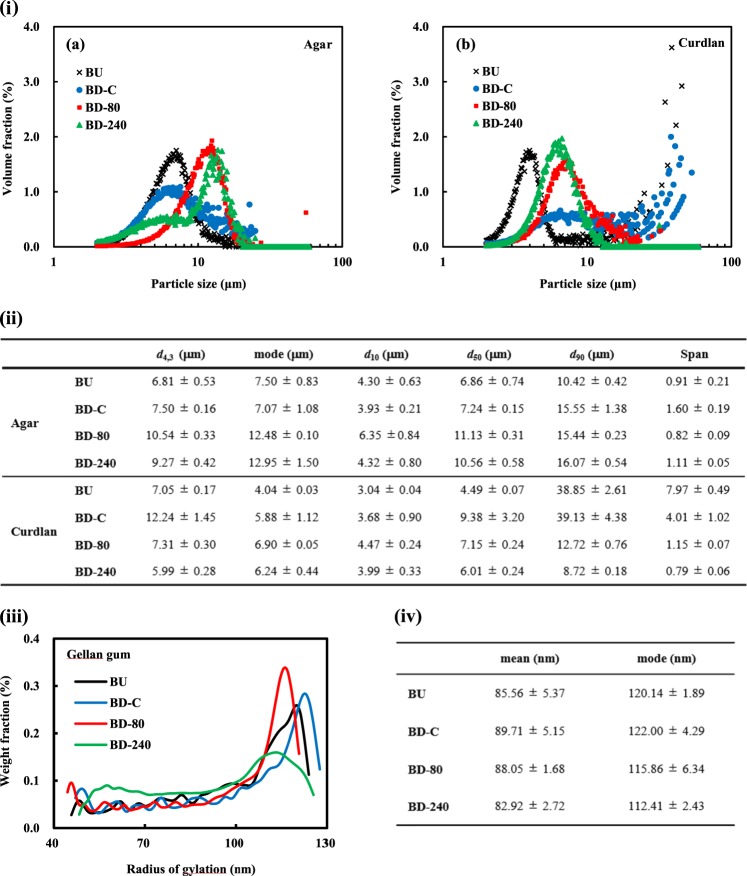
Particle size analyses of the prepared microgels. **i**-a, **i**-b Volume-weighted particle size distribution of agar and curdlan microgels measured by Coulter principle particle size analyzer with a detection range of from 2 to 60 μm. **ii** Representative values calculated from the particle size distributions of agar and curdlan microgels (mean ± S.D., *n* = 3). **iii** Weight-based particle size distribution of gellan gum microgels analyzed by SEC-MALS. **iv** Mean and mode sizes of gellan gum microgels calculated from the particle size distributions (mean ± S.D., *n* = 3)

**Fig. 3:**
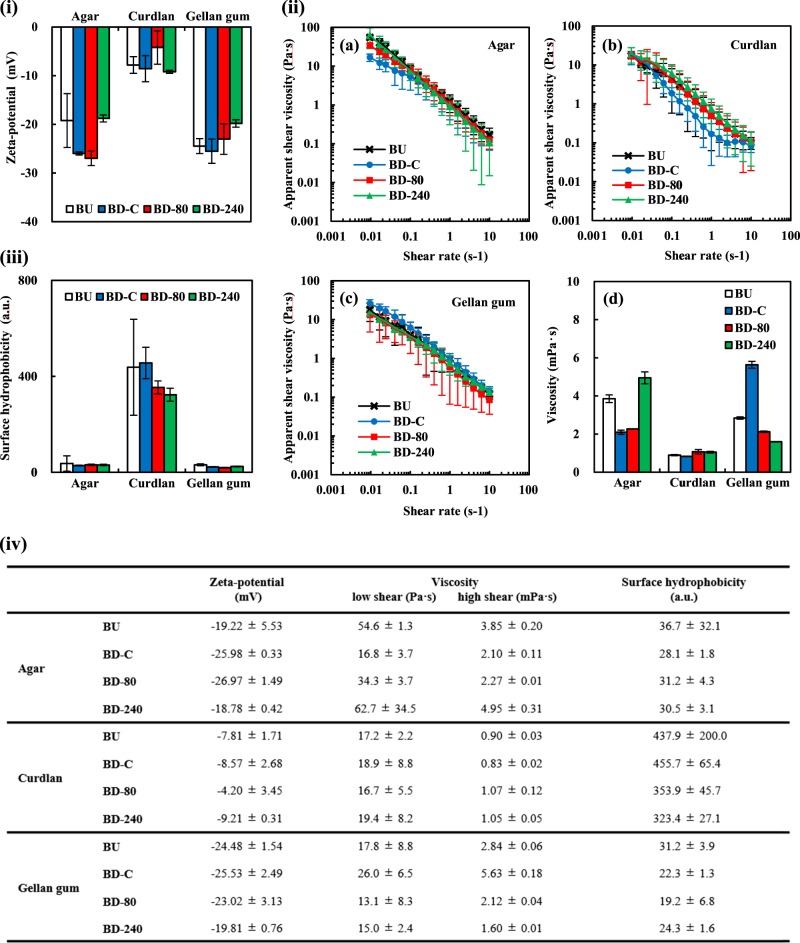
Colloidal properties of the microgel suspensions (mean ± S.D., *n* = 3). **i** Zeta-potential of microgel suspensions in an aqueous phase at 25 °C. **ii**a–c Shear rate-dependent changes of apparent shear viscosity of microgel suspensions (0.125 wt%) measured using the rotational viscometer with a coaxial dual cylinder at 25 °C. **ii**-d Viscosity measured by vibration viscometer with a vibration amplitude of 0.4 mm. **iii** Surface hydrophobicity analyzed with ANS-Na. The slope of fluorescence intensity (excitation and emission wave length of 390 and 470 nm, respectively) vs polysaccharide concentration biplot was linearly calculated as a surface hydrophobicity index. **iv** Summarized values of zeta-potential, viscosity, and surface hydrophobicity analyses. The viscosity values were obtained by rotational viscometry at the low shear rate of 0.0096 s^−1^ (left) and by vibration viscometry at the high shear rate of >10 s^−1^ with a vibration amplitude of 0.4 mm. All the values are expressed as mean ± S.D. (*n* = 3)

Figure 2i-a and i-b shows volume-based particle size distributions of the agar and curdlan microgels measured by the Coulter principle-based particle size analyzer. Representative values from the distributions are summarized in Fig. 2ii. The particle size distribution of BU, BD-C, and BD-80 agar microgels was monomodal, whereas that of BD-240 seemed to be bimodal (Fig. 2i-a). The average size of BU and BD-C particles without the high-pressure treatments was smaller than that of BD-80 and BD-240 ones with the treatments according to the *d*_4,3_ and mode values shown in Fig. 2ii. It should be noted that the average particle sizes rather increased via high-pressure processing that is usually helpful for obtaining finer particles. This unexpected contrary tendency is probably attributed to partial solubilization of agar particles by the ultra-high-pressure treatments and subsequent reorganization leading to spontaneous formation of large particles. The partial solubilization can be supported by the presence of fibrous structure observed by cryo-SEM marked with * (Fig. 1a4) and the increased viscosity presented in the following section (Fig. 3ii-a, ii-d). The Span value as an index of polydispersity obtained from the size distribution of the agar microgel particles was significantly different from each other (one way ANOVA, *p* < 0.05), while there were no clear relationships between the Span values and the added shear stresses during the sample preparation (Fig. 2ii).

The particle size distributions of all types of the curdlan particles mostly seemed to be monomodal with almost the same size range as that of the agar particles approximately from 2 to 20 μm (Fig. 2i-b). The BU suspension with an averaged particle size of 4 μm included relatively larger particles >20 μm probably originated from the water-insoluble powdered curdlan that could not be completely dispersed to the aqueous phase by high-speed blending of the curdlan powder. The BD-C suspensions prepared via the formation of macrogels also possessed such large particles, whereas the particles of approximately 6 μm with a broader monomodal distribution were predominantly observed. The large particles observed for the BU and BD-C suspensions extinguished through the high-pressure processing applied for the BD-80 and BD-240 samples, meaning that most of them were successfully atomized into micron-ordered particles by the processing. The resultant distributions obtained from the BD-80 and BD-240 were both monomodal with the mode values of 6.90 and 6.24 μm, respectively. On the contrary to the Span values calculated from particle size distributions of the agar microgels, the values of the curdlan microgels decreased depending on the increase of the added shear stresses during the breaking-down treatments.

On the other hand, due to the limited detection range (2–60 μm) of the used aperture unit, it was difficult to determine the particle size distributions of the gellan gum microgels, indicating that the particle size of most populations were below 2 μm. In fact, the gellan gum microgels easily passed through a filter with 2 μm pores without any excessive pressure and were difficult to find in the cryo-SEM images (Fig. 1c1–c4). To analyze the particle size distribution of gellan gum microgels, SEC-MALS was further performed for the filtrated gellan gum suspensions. Figure 2 iii shows differential particle size distributions of radius of gyration obtained from the SEC-MALS for the gellan gum microgle suspensions. All the size distributions looked like the normal distribution, different from the log-normal distribution typically observed in a volume-weighted laser diffraction particle size analysis. The predominant peaks were present between 100 and 130 nm for all the samples (Fig. 2iii) and the average sizes were around 100 nm (Fig. 2iv), clearly demonstrating that the gellan gum microgels were nano-ordered assemblies. According to Takahashi et al.^31^ the average radius of solubilized gellan gum measured by static light scattering at 40 °C were about 30 nm; therefore, we can conclude that we were able to prepare gellan gum microgels throughout our methods. This can be supported by the fact that the major peak area of particle size distributions of the BD-C gellan gum microgels obviously decreased and subsequently a novel peak appeared at around 55 nm after the high-pressure treatment at 240 MPa (BD-240), presumably due to additional dissociation between gellan gum molecules forming microgels.

To estimate interactive forces of a dispersed microgel particle between other surrounding particles or the surface of added oils during the emulsification in the following process, zeta-potential of microgel particles were determined (Fig. 3i). There was statistically significant difference between the zeta-potential values of the agar microgel particles prepared via the various types of processing (one-way ANOVA, *p* < 0.05). The obtained zeta-potential values of the agar microgel particles were similar to those reported in previous studies, that is approximately −20 mV.^32,33^ The BD-C and BD-80 particles prepared via macrogel formation indicated slightly lower zeta-potential magnitudes, which could be attributed to different structural features related to the surrounding state of sulfated groups on the surface of particles. All the absolute zeta-potential values were of about 20 mV, probably enough to generate electrically repulsive forces between particles helpful for preventing strongly binding aggregation.^34,35^ The results also suggest that the negatively charged agar particles would not preferably access to the oil surfaces with negative charge by hydroxyl ions which are newly created during emulsification.^28,36,37^

The zeta potential values of curdlan microgels were not significantly different among all the samples, and the magnitudes of the curdlan microgel particles were totally <10 mV and relatively smaller compared to that of the agar ones presumably because curdlan molecules mostly consist of β-glucan without any acidic groups. The low zeta-potential values generally lead to weak repulsive forces between colloidal particles, maybe explaining the presence of large granules detected in the particle size analyses of the curdlan BU and BD-C (Fig. 2i-b) and suggesting that the dispersed curdlan microgel particles tend to associate to form weakly-packed flocs. During emulsification with added oils, it is expected that the curdlan microgels are able to more easily access to oil-water interfaces than the agar microgels regarding electrostatic interactions, contributing to efficient adsorption onto the newly created oil droplet surfaces.

In the case of gellan gum, all the zeta-potential values of the microgels seemed to be independent of the processing during the microgel preparation. The zeta-potential values of the gellan gum microgels were generally similar to those of the agar microgels according to the presence of carboxyl groups,^22^ implying that the gellan gum particles may not attractively interact with each other and with negatively charged oil surfaces newly created during emulsification as discussed for the agar microgels.

To shed insight into the assembly structure of polysaccharide microgel macromolecules in aqueous phases and to assess adsorption efficiency to the oil–water interfaces via diffusion process to form microgel-stabilized emulsions in the following section, viscosity of the microgel suspensions was measured using the coaxial-cylinder rotational-type viscometer suitable for flow behavior analyses and low shear rates measurements and tuning-fork vibration viscometer appropriate for low viscosity liquid as water at high shear rates (Fig. 3ii). Since flow behavior of all the microgel suspensions were non-Newtonian and typical shear-thinning (Fig. 3ii-a–c), we discuss representative values at a low shear rate (0.0096 s^−1^) and a high shear rate (vibration amplitude = 0.4 mm that is equivalent to >10 s^−1^) obtained from the two viscometers.

The viscosity of all the microgel suspensions at the low shear rate was extremely higher than that of water only about 0.98 mPa·s at 25 °C (Fig. 3ii-a), clearly indicating that there were some attractive interactions between the dispersed microgel particles regardless of the polysaccharide types. The viscosity of agar microgel suspensions at the low shear rate was significantly different according to the microgelation methods (one-way ANOVA, *p* < 0.05) and the values were in order of BD-240 ≈ BU > BD-80 > BD-C. This tendency corresponds to possible partial dissociation states of agar macromolecules obtained from the cryo-SEM observation; i.e., the outside fibrous network of the microgel particles expanded into the surrounding aqueous phase particularly for BU and BD-240 samples (marked by * in Fig. 1a1, a4). Therefore, the different viscosity values can be explained by overlapping, inter-penetrating, and entangling of polysaccharide macromolecules via the frequently switching hydrogen bonding.^38^

The viscosity of curdlan microgel suspensions was not significantly different among the processing at the low shear rate (Fig. 3ii-b). All the curdlan microgel suspensions were of relatively low viscosity to the agar suspensions at the same shear rate (Fig. 3ii-a, ii-b). Throughout the cryo-SEM analyses (Fig. 1b1–b4), we confirmed that the curdlan microgel particles seemed to be independently dispersed without clearly observed expanding fibrous networks in the aqueous phase. These results mean that curdlan microgel particles more weakly interacted attractively with each other in water compared to agar microgels, regardless of the relatively low zeta-potential magnitudes of curdlan microgels leading to particle aggregations. Similar tendencies were found for the differently treated gellan gum microgel suspensions (Fig. 3ii-c) and for the overall viscosity values of the gellan gum microgel suspensions in comparison with the agar suspensions (Fig. 3ii-a, ii-c).

The shear viscosity values obtained by the rotational viscometer at the relatively high shear rates were inevitably accompanied with nonnegligible experimental errors due to the detection limit. We therefore performed high shear rate vibro-viscometry and subsequently obtained viscosity values (Fig. 3ii-d). At the high shear rate, the viscosity of agar microgel suspensions was significantly different according to the microgelation methods (one-way ANOVA, *p* < 0.05) and the values were in order of BD-240 > BU > BD-80 ≈ BD-C. All the curdlan microgel suspensions were of relatively low viscosity at almost the similar level to that of water only (about 0.98 mPa·s at 25 °C), and less viscous than the agar suspensions. The viscosity of gellan gum microgel suspensions was significantly different (one-way ANOVA, *p* < 0.05), and it was highest for BD-C, followed by BU, BD-80, and BD-240. All these tendencies were similar to the results obtained by the high shear rate rotational viscometry.

### Emulsifying properties of microgel particles

#### Emulsifying ability

First of all, in order to examine emulsifying ability of the polysaccharides themselves, agar, curdlan, and gellan gum were dissolved into water and homogenized with soybean oils to produce soybean oil-in-water emulsions as described in Materials and methods. In this study, we defined emulsifying ability as whether or not 20 wt% of soybean oils—the oil content applicable for various commercial food products—can be completely emulsified into fine oil droplets in the colloidal scale ranged between 10^−6^ and 10^−9^ m. Even though the preparation methods were intensively oriented to make the appropriate polysaccharide solution so that the temperature, pH, and ionic strength conditions were not necessarily controlled completely, i.e., not directly comparable to the microgel suspensions, we confirmed that the temporarily emulsified oils extensively turned into bulk states in a macroscopic scale immediately after homogenization processes (data not shown).

To reveal effects of microgelation on the emulsifying ability of the polysaccharides, we prepared polysaccharide microgel suspensions to create microgel-based emulsions containing the same oil, followed by visual observations of initial states of the emulsions. Resultantly, all the agar and curdlan microgel-stabilized emulsions did not macroscopically separate into oil and water phases at 25 °C, indicating that the agar and curdlan microgels possess high emulsifying ability worthwhile thoroughly investigating the emulsifying properties in the following section. On the other hand, the gellan gum microgel suspensions could not stabilize all the added oils in the colloidal scale with free oil layers present at the top.

Emulsifying ability is often discussed by interfacial tension measurements for classical small-molecule emulsifier-stabilized emulsions, whereas the tensiometry is not necessarily applicable for particle-stabilized emulsions due to the limited capability of particles to reduce the interfacial tension between oil and water.^39^ Although the adsorption schemes of soft microgels at the interface are still in arguments,^29^ we confirmed that the polysaccharide microgels prepared in this study scarcely lowered the interfacial tension at the oil–water interface according to the pendant drop tensiometry (data not shown), probably indicating the tensiometry is not a useful tool for discussing emulsifying ability of the surface-inactive polysaccharide microgels.

Emulsifying ability of a surface-active molecule is generally recognized as the resultant balance of two-competently occurring coincide phenomena—formation and re-coalescence of oil droplets during emulsification process consisting of adsorption efficiency and resistance against oil droplet collisions. Adsorption efficiency related to availability of applied shear energy depends on accessibility and diffusion rate of the surface-active molecule to the newly created oil surfaces. These molecular properties for the three kinds of microgel samples were estimated by the zeta-potential, viscosity at the high shear rate, and particle size analyses (Figs. 2 and 3i, ii-d). As a result, the accessibility represented as zeta-potential was in order of curdlan > agar ≈ gellan gum, and the diffusion rate assessed by viscosity and particle size was curdlan > agar ≈ gellan gum and gellan gum > agar ≈ curdlan, respectively. In addition, since adhesiveness or affinity of the surface-active molecule to oil phases often represented by surface hydrophobicity could affect its adsorption efficiency, we further evaluated the surface hydrophobicity of microgel particles using a fluorescence probe, ANS-Na. The respective obtained surface hydrophobicity index values of BU, BD-C, BD-80, and BD 240 microgel particles were 36.7 ± 32.1, 28.1 ± 1.8, 31.2 ± 4.3, and 30.5 ± 3.1 for agar, 437.9 ± 200.0, 455.7 ± 65.4, 353.9 ± 45.7, and 323.4 ± 27.1 for curdlan, and 31.2 ± 3.9, 22.3 ± 1.3, 19.2 ± 6.8, and 24.3 ± 1.6 for gellan gum (Fig. 3iii), meaning that the adhesiveness or affinity evaluated based on surface hydrophobicity was curdlan > agar ≈ gellan gum. All these tendencies do not totally correspond to the emulsifying ability of polysaccharide microgels, that is agar ≈ curdlan > gellan gum, suggesting the adsorption efficiency that is critically important at the initial stage of emulsion formations ought not to be of decisive significance for the emulsifying ability.

It should be noted that large microgels prepared with agar and curdlan were able to stabilize all the added oils, whereas small-sized gellan gum microgels were not capable of emulsifying all the added oils. In addition to this, the free oil volume ratio to the added total oils of BU, BD-C, BD-80, and BD-240 was 20.8%, 32.3%, 54.1%, 93.2%, respectively; this is correlated to the mode values of particle size distribution of gellan gum microgels (*r* *=* −0.929). These results suggest that the particle size of microgels plays a critical role in successful creation of soybean oil-in-water emulsions stabilized by various polysaccharide microgels presumably because large particles adsorbed on the oil–water interface to form thick interfacial layers effectively preventing re-coalescence of created oil droplet surfaces during the emulsification processes. The successful emulsification observed above probably argues that agar and curdlan microgels adsorb to the oil-water interface as described for microgels prepared with other materials like inorganic polyacrylamide and edible proteins.^27,28^ In the following parts, we investigated emulsion properties for the agar and curdlan microgels.

**Fig. 4:**
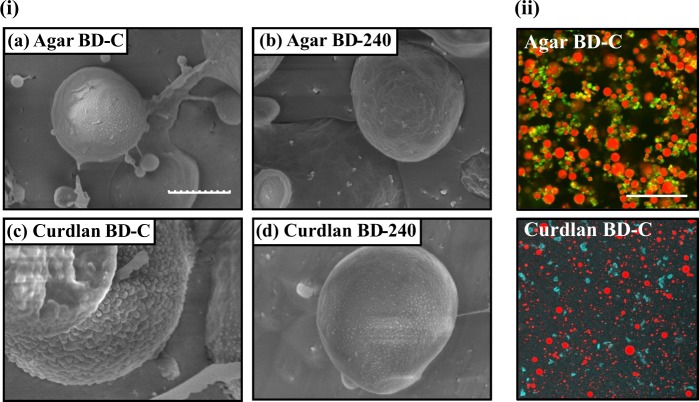
Microstructure of the microgel-stabilized emulsions. **i** The oil droplet surfaces of microgel-stabilized emulsions observed by cryo-SEM. Scale of all images in this part is the same (scale bar = 1 μm). **ii** CLSM images of the microgel-stabilized emulsions stained by Nile Blue A and Nile Red for agar-based ones, and ANS-Na and Nile Red for curdlan-based ones, respectively. The excitation and emission wave lengths of the dyes were 635 and 647 nm for Nile Blue A, 405 and 422 nm for ANS-Na, and 473 and 520 nm for Nile Red, respectively. Scale of both images in this part is the same (scale bar = 100 μm)

To confirm the successful adsorption of microgel particles onto the oil droplet surfaces, the agar and curdlan microgel-stabilized emulsions were observed by cryo-SEM. Since similar images were obtained for BU, BD-80, and BD-240 emulsions made with agar and curdlan microgels, images of BD-240 and BD-C emulsions are presented in this section (Fig. 4i). The oil droplet surfaces of agar BD-C and BD-240 emulsions seemed to be covered with the macromolecules, whereas those of the BD-C emulsions were slightly different from those of BD-240 ones (Fig. 4i-a, b). The different structure cannot be necessarily attributed to the particle size of agar microgels as shown in Fig. 2. Compared to the oil droplet surfaces of agar microgel-based emulsions (Fig. 4i-a, b) and curdlan BD-240 microgel-based emulsions (Fig. 4i-d), those of curdlan BD-C microgel-based ones were likely surrounded by much larger microgel particles (Fig. 4i-c). It is possible that only BD-C samples prepared via the formation of macrogels without ultra-high-pressure treatments had such a unique structure.

**Fig. 5:**
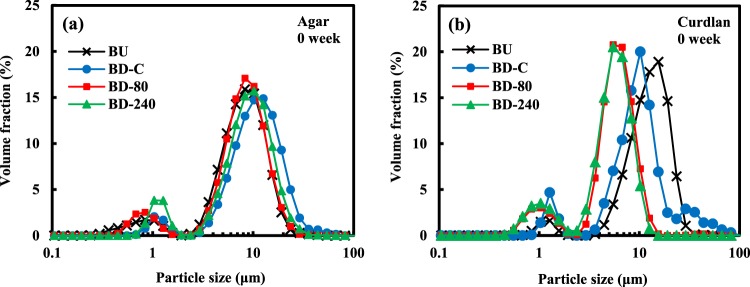
Particle size distribution of the oil-in-water emulsions stabilized by microgels prepared with **a** agar and **b** curdlan. Measurements were carried out under the presence of SDS using a laser-diffraction particle size analyzer with a refractive index of 1.45–0.00*i* (*n* = 3)

The particle size distribution of the agar and curdlan microgel-stabilized emulsions was measured by a laser-diffraction particle size analyzer after diluting with deionized water including SDS that is helpful for excluding oil droplet aggregation and clarifying actual oil droplet size.^40^ The oil droplet size distributions of all the samples did not significantly change regardless of the presence of SDS, indicating that the oil droplets did not strongly interact with each other to form aggregates under the tested conditions. Figure 5a presents the distributions of all the agar-based emulsion oil droplets prepared by the microgels fabricated via varied processing. They were generally all bimodal and the major peak was at around 10 μm. This tendency was observed for the curdlan-based emulsion oil droplets as well with a slight variation in the main peak (Fig. 5b). The calculated mean value, *d*_4,3_ of BU, BD-C, BD-80, and BD-240 emulsions was 6.07 ± 1.11, 8.27 ± 2.89, 7.21 ± 2.35, and 7.20 ± 2.03 μm for agar and 11.03 ± 2.50, 8.66 ± 1.03, 5.00 ± 0.47, and 4.61 ± 0.50 μm for curdlan, respectively.

#### Emulsion stability

**Fig. 6:**
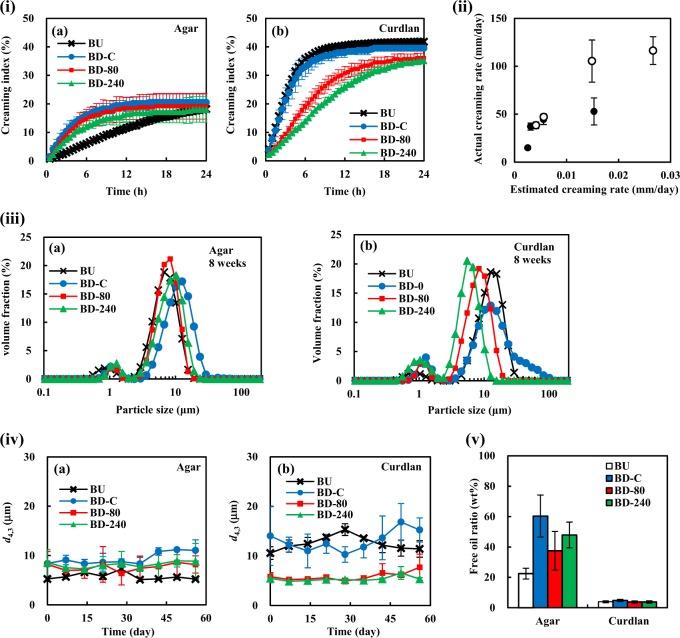
Physical stability of the microgel-stabilized emulsions (mean ± S.D., *n* = 3). **i** Time-dependent change of creaming indices for the a agar and b curdlan microgel-stabilized emulsions. Experiments were carried out at 20 °C for 24 h. **ii** Correlation of estimated and actual creaming rates of the microgel-stabilized emulsions. The estimation was performed based on the Stokes law using viscosity values of the corresponding continuous phases at the low shear rate (0.0096 s^−1^) and the mean sizes of the emulsion oil droplets. The filled and open symbols indicate the values of agar- and curdlan-based emulsions, respectively. **iii** Particle size distributions of a agar microgel- and b curdlan microgel-based emulsions after 8 weeks storage at 25 °C. **iv** Periodical change of *d*_4,3_ of the microgel-based emulsions measured using the particle size analyzer during storage test at 25 °C. **v** Amount of free oils forcibly released from the microgel-based emulsions by centrifugation at 20,000×*g* for 30 min at 25 °C. The determination was conducted using Oil Red O according to Palanuwech et al.^49^

To evaluate emulsion stability against creaming, periodical macroscopic observations were carried out to calculate creaming index of the prepared emulsions. Figure 6i-a shows time-dependent changes of creaming index for the agar microgel-stabilized emulsions for 24 h static storage at 20 °C. The creaming index of agar-based emulsions reached plateau after 24 h storage, but the creaming rate at the initial stage was much slower for BU samples than that for BD samples depending on the microgel preparation methods. On the contrary, reverse trends for the creaming rate with regard to the microgel processing were found as for curdlan-based emulsions (Fig. 6i-b). The final creaming indices of agar and curdlan samples were of about 20% and 40%, respectively.

Based on the obtained results on *d*_4,3_ of the emulsion oil droplets and viscosity of the continuous phase at the low shear rate shown above, we calculated theoretical initial creaming rate values of the samples using the Stokes law assuming that there are not any interactions between oil droplets which induce an increase of effective hydrodynamic radius of emulsion oil droplets. The used viscosity values of the BU, BD-C, BD-80, and BD-240 microgel suspensions were 54.6 ± 1.3, 16.8 ± 3.7, 34.3 ± 3.7, and 62.7 ± 34.5 Pa·s for agar and 17.2 ± 2.2, 18.9 ± 8.8, 16.7 ± 5.5, and 19.4 ± 8.2 Pa·s for curdlan, respectively (Fig. 3ii-a, b). Figure 6ii is a biplot graph of the observed initial creaming rates and the theoretical ones introduced by the Stokes law. The results show that the observed values were extremely higher than the theoretical values, demonstrating that the initial creaming rate of both agar and curdlan samples does not satisfy the assumption of the Stokes law, i.e., the emulsion oil droplets did considerably form flocculates that quickly cream, regardless of the large magnitudes of zeta-potential of the emulsion oil droplets; that is, the values of BU, BD-C, BD-80, and BD-240 emulsions were −31.58 ± 5.71, −28.01 ± 2.93, −32.45 ± 1.43, and −34.83 ± 1.76 mV for agar and −32.38 ± 1.04, −35.93 ± 2.62, −37.23 ± 6.63, and −38.61 ± 7.79 mV for curdlan, respectively. This can be supported by microstructure of the microgel emulsions shown in Fig. 4ii. The molecular dynamics of microgels on the oil droplet surfaces and in the continuous phase such as overlapping etc. may have caused the significant flocculation.

As well as the initial creaming behavior, the final values of creaming index reached at the end of the measurements were clearly different between agar and curdlan samples (Fig. 6i-a, b), suggesting that the packing states of the emulsion oil droplets were different between agar and curdlan samples. In order to clarify the existing state of oil droplets in the continuous aqueous phase, microgel-based emulsions were prepared with fluorescence dyes to differentiate oil droplets and microgels by laser excitation in the confocal laser scanning microscopy (CLSM) observations (Fig. 4ii). In general, the oil droplets of all the agar emulsions were packed less densely than those of all the curdlan emulsions, whilst the packing state seemed to be similar among the differently-treated microgel-based emulsions. The agar microgels appeared to be abundantly present among oil droplets to form a co-existing three-dimensional network, probably helpful for preventing the droplets from approaching each other that leads to no further creaming. Kaneda previously reported that agar microgel suspensions required small yield stresses to flow,^41^ indicating that such a network should exist in the agar microgel-based emulsions we prepared.

To test emulsion stability against oil droplet coalescence during long-term static storage, time-dependent particle size measurements were performed by a laser diffraction particle size analyzer. Figure 6iii, iv shows the particle size distributions and the periodical change of volume-weighted mean particle diameter, *d*_4,3_ of the samples. The particle size of all the emulsions did not considerably change for 8 weeks (Figs. 5 and 6iii, iv), obviously indicating that both the agar and curdlan microgel-based emulsions are extremely stable against oil droplet coalescence for long time under static conditions. The significantly high stability of the sample emulsions against coalescence was typically known for Pickering and Mickering emulsions and agrees with other previous reports.^28,42^ Meanwhile, we measured the released free oils at the top after centrifugal acceleration of emulsion destabilization (Fig. 6v). The amount of released oils from agar microgel-based emulsions was much larger than that from curdlan-based emulsions. This can be attributed to the different surface hydrophobicity that is related to the adsorption forces of microgels to the oil–water interface. That is, a more hydrophobic region of curdlan microgel particle surface is able to penetrate oil droplets, more strongly anchoring microgel particles to oil droplets. Such strongly-bound microgels of curdlan may be resistant to the removal from oil droplets by the high speed centrifugation, thereby keeping thick layer to prevent coalescence.

## Discussion

In this study, we investigated effects of microgelation on emulsifying ability of agar, curdlan, and gellan gum that have been scarcely utilized as stand-alone emulsifying agents. Agar and curdlan acquired the emulsifying ability via microgelation, whereas gellan gum did not. The averaged oil droplet size of the obtained agar and curdlan microgel stabilized emulsions was almost similar and totally independent of the preparation methods, i.e., bottom-up or top-down approaches. Among the major physical properties of microgel particles, we can conclude that particle size of microgels particularly plays a critical role in stabilizing oil-in-water emulsions. The creaming stability of the obtained emulsions was significantly different depending on not only the kinds of polysaccharides but also the microgelation methods. All the emulsions were stable against oil droplet coalescence under the static condition, while the agar microgel-based emulsions destabilized under the accelerated dynamic condition, different from the curdlan microgel-based ones. We are able to attribute the different behavior to the surface hydrophobicity of agar and curdlan microgels relating to solubilization of the fiber chains into the dispersed oil phases.

The surface hydrophobicity of agar and curdlan molecules is presumably originated from the carbon backbone and side chains with less polar groups of branched agar and the backbone of non-branched linear curdlan, in relation to the number of effective polar hydroxyl groups exposed to the water phase in an aqueous solution. Conversion of dispersed agar and curdlan polysaccharide molecules to microgels may have decreased the number of exposed hydroxyl groups throughout formation of intermolecular hydrogen bonding or molecular conformational changes due to the helices associations, that leads to the increased surface hydrophobicity affecting the emulsion stability. Independent of the hydrophobicity, we must again emphasize that emulsifying ability can be achieved by the micron-ordered microgels providing thick adsorbed layers on the oil droplet surfaces.

## Materials and methods

### Materials

Agar, curdlan, and gellan gum were purchased from FUJIFILM Wako Pure Chemical Corporation, Osaka, Japan. The typical average molecular weight of agarose and agaropectin (components of agar), curdlan, gellan gum is about >100, <20, 66–680, and 250–490 kDa, respectively.^21,23,43,44^ Soybean oil was also obtained from the same company. Oil red O, Nile Red, and Nile Blue A were bought from MilliporeSigma, Burlington, MA, USA. Sodium 9-anilino-1-naphthalenesulfonate (ANS-Na) was acquired from Tokyo Chemical Industry Co., Ltd., Tokyo, Japan. All other general chemicals were of analytical grade. Deionized water was used for preparation of all samples and aqueous solutions.

### Sample preparation

#### Preparation of microgel suspensions

##### Building-up methods

Agar powder (0.125 g) was dispersed to deionized water and heated at 95 °C for 30 min to obtain 100 g of the solutions. The solution was cooled in a water bath at 25 °C for 30 min, mixing at 550 rpm by a magnetic stirrer to prevent sedimentation and partial macroscopic gelation during this process. Curdlan powder was mixed with deionized water by a high-speed blender (Physcotron, Microtec Co., ltd, Chiba, Japan) with NS-10 shaft at 20,000 rpm for 3 min at room temperature, followed by the deaeration for 10 min. The suspension (100 g, 0.125 wt%) was incubated in a water bath first at 95 °C for 30 min and subsequently at 25 °C for 30 min, mixed by the stirrer at 550 rpm during both the heating and cooling processes. Gellan gum powder (0.125 g) was dissolved to deionized water at 95 °C for 30 min. The solution (95 g) was blended with 5 g of 10 mM calcium chloride solution, and then mixed at 550 rpm by the stirrer for 30 min at 25 °C. These samples prepared via the building-up processes were referred to as BU. The pH value of all the samples was at around 6.0 with a maximum variation of 0.3.

##### Breaking-down methods

Macrogels made from agar, curdlan, and gellan gum were prepared in different ways as follows. For agar macrogels, agar powder was mixed with deionized water by a magnetic stirrer at 550 rpm for 10 min to obtain 1.0 wt% of agar suspensions. The suspension (100 g) was heated in a water bath at 95 °C for 30 min to obtain an agar solution and subsequently cooled in another water bath at 25 °C for 30 min to achieve a cold-set macrogel formation. For preparing curdlan macrogels, 1.0 g of curdlan powder was homogenized with 39 g of deionized water by the high-speed blender as stated above. The suspension was deaerated using a vacuum pump for 10 min and subsequently mixed with 60 g of additional hot water (95 °C), followed by an incubation in a water bath at 95 °C for 30 min to ensure heat-set gelation.^23^ The obtained macrogel was cooled down to room temperature in another water bath (25 °C, 30 min). For gellan gum, 1 g of gellan gum powder was dispersed in 79 g of deionized water and incubated at 95 °C for 30 min to obtain a gellan gum solution. The solution was mixed with 20 g of a 10 mM calcium chloride solution and placed in a water bath at 25 °C for 30 min to set an ion-induced macrogel.

Each macrogel was roughly broken into small pieces by a spatula and mixed with additional deionized water to obtain 400 g of a macrogel suspension, and subjected to homogenization by the high-speed blender with NS-20 shaft at 15,000 rpm for 1 min at room temperature to obtain breaking-down coarse microgel (BD-C) suspensions. The BD-C suspensions were further processed through a high-pressure homogenizer (Star Burst Mini, Sugino machine limited, Toyama, Japan) at 80 or 240 MPa to make fine microgel suspensions. These suspensions were defined as breaking-down samples with various high-pressure treatments, i.e., BD-80 and BD-240. All the microgel suspensions were immediately twice diluted with deionized water to achieve the final polysaccharide concentration of 0.125 wt%. The pH value of all the BD-C, BD-80, and BD-240 samples was also at around 6.0 with a maximum variation of 0.3.

#### Emulsion preparation

The microgel suspensions (40 g) were mixed with soybean oils (10 g) in a plastic bottle by the high-speed blender with NS-20 shaft (15,000 rpm, 1 min) at room temperature, and the resultant coarse emulsions were processed in an ice-water bath by an ultrasonic homogenizer (Q700 Sonicator, Qsonica, L.L.C, Newtown, CT, USA) with a 1/2 inch probe at an amplitude of 30% for 1 min to make fine microgel-based emulsions. Control emulsions were also prepared by the following polysaccharide solutions without the gelation processes. Agar solutions (0.125 wt%) melted at 95 °C were incubated in a water bath at 60 °C ensuring solubilized states,^21^ and then homogenized with soybean oils via the same homogenization processes at a weight ratio of 1:4 in a bottle incubated in the water bath at 60 °C. The obtained emulsions were kept at 60 °C to prevent gelation of agar.^21^ Curdlan (0.125 wt%) was dissolved into a 0.2 M sodium hydroxide solution at about pH 13.0 and homogenized with soybean oils as described above. Gellan gum (0.125 wt%) was solubilized in distilled water without any additional salts at 95 °C and cooled down to 25 °C in a water bath, and then homogenized in the same way. Visual observation for all the emulsions was carried out for 24 h to confirm free oils released from the emulsions.

### Cryo-scanning electron microscopy (Cryo-SEM)

The microgel suspensions and microgel-stabilized emulsions were observed by a scanning electron microscope (SU8230, Hitachi High-Technologies Corporation, Tokyo, Japan) equipped with a cryogenic unit (Alto2500, Gatan UK, Abingdon, Oxfordshire, UK). The samples were filled into two metal hollow rivets with the edges contacted, and rapidly frozen in nitrogen slush. The lower part of the rivets was fixed on a metal stage and then inserted into the loading chamber of the microscope, and subsequently split into the original two parts to remove the upper one. The cleaved surface of the lower rivet was observed after appropriate sublimation at −120 °C.

### Particle size analysis

Three methods were applied for particle size analysis of microgel suspensions. The particle size distribution of microgel suspensions was first analyzed by dynamic light scattering (DLS) at 25 °C (Nicomp 370 submicron particle size analyzer, Particle Sizing System, Inc., Port Richey, FL, USA) using the solid particle mode and Nicomp distribution analysis mode assuming multi-modal distributions.

The particle size of the microgel suspensions was also measured using a laser-diffraction particle size analyzer (SALD 2200, Shimadzu Corporation, Kyoto, Japan) with the refractive index of 1.45–0.00*i*.

Next, the particle size distribution of microgel suspensions was determined using a Coulter counter particle size analyzer (Multisizer 4e, Beckman Coulter, Brea, CA, USA). An aliquot of the sample suspensions containing 0.125 wt% of polysaccharides were dispersed into a 0.9 wt% sodium chloride solution in a dispersing chamber with an assistance of 0.2 wt% SDS that prevent possible aggregation. The volume-based particle diameter is individually calculated from conductivity change between the electrodes in an aperture unit. A 100 μm aperture unit with the detection range of from 2 to 60 μm was used, and the particle size distribution was obtained from ~50,000 particles. The *d*_4,3_, mode, *d*_10_, *d*_50_, and *d*_90_ values were obtained from the distribution, and SPAN was calculated according to the following equation:^45^

1 $${\mathrm{SPAN = }}\frac{{d_{{\mathrm{90}}} - d_{{\mathrm{10}}}}}{{d_{{\mathrm{50}}}}}$$

For the gellan gum microgel suspensions, the fourth method, i.e., size exclusion chromatography coupled with multi-angle light scattering (SEC-MALS) was applied for determining particle size distribution. The gellan gum microgel suspensions (0.25 wt%) were filtrated through a syringe-driven disposable filter unit with a polypropylene membrane with 2 μm pore (Puradisc™ 25 GD2, Whatman®, Whatman Inc., Marlborough, MA, USA). The filtrates (50 μl) were injected and separated by a size exclusion chromatograph (1260 Infinity, Agilent Technologies, Santa Clara, CA, USA) through three columns (OHpak SB-G 6B guard column, OHpak SB-806 HQ, and OHpak SB-805 HQ, Shodex™, Showa Denko K.K., Tokyo, Japan) at the flow rate of 0.5 ml min^−1^. The separated samples were subsequently detected by a UV absorbance detector (1260 VWD, Agilent Technologies, Santa Clara, CA, USA) at 258 nm, a differential refractive index detector (Optilab T-rEX, Wyatt Technology Corporation, Santa Barbara, CA, USA) with dn/dc value of 0.155 ml mg^−1^,^44^ and multiple-light scattering detectors (DAWN HELEOS-II Wyatt Technology Corporation, Santa Barbara, CA, USA). The obtained data were processed using an attached software (ASTRA Ver. 6.1) to calculate radius of gyration of particles and its weight-based distribution. The concentration of samples was assumed to be 2.5 mg ml^−1^, and a 0.1 M NaNO_3_ solution was used as an eluent. All the analysis were carried out at 25 °C.

The microgel-based emulsions were subjected to particle size analysis using the laser-diffraction particle size analyzer in the same way as the measurements of microgels. Volume weighted mean particle size (*d*_4,3_) was calculated for presentation.

### Zeta-potential measurements

The zeta-potential of microgel particles and emulsion oil droplets was measured using a laser-Doppler zeta-potential analyzer (ELS-Z1, Otsuka Electronics C. ltd., Osaka, Japan). The microgel suspensions and microgel-based emulsions were several times diluted with deionized water to obtain appropriate light scattering intensity when required. The measurements were performed at 25 °C and repeated three times for each sample.

### Rheological measurements

#### Rotational viscometry

Apparent shear viscosity of the microgel suspensions was measured using a rotational rheometer (Rheosol G3000, UBM, Kyoto, Japan) attached with a coaxial-double-cylinder probe. An aliquot (3.0 ml) of samples incubated in a water bath at 25 °C was poured into the lower outer hollow cylinder (*ϕ* = 17.99 mm), and subsequently the cylindrical part of the upper inner cylinder (*ϕ* = 15.99 mm, h = 26.04 mm) was completely immersed into the sample to adjust the gap between these cylinders’ bottoms to 6 mm. The lower outer cylinder was rotated and the shear rate dependent changes of the shear stress were measured within a range from 0.0096 to 9.97 s^−1^ at 25 °C. Apparent viscosity values were calculated using obtained shear stress (*σ*) and shear rate ($\dot \gamma$) values based on the following equation:^46^

2 $${\mathrm{Apparent}}\,{\mathrm{viscosity = }}\frac{\sigma }{{\dot \gamma }}$$

#### Vibration vicometry

The viscosity measurement for microgel suspensions was carried out using a tuning fork vibro rheometer (RV-10000A, A&D company, limited, Tokyo, Japan). A 10 ml of sample was subjected to the measurements with 0.4 mm of amplitude for 3 min to collect static viscosity (*V*_s_) converted from the vibration resistance. The viscosity of the samples was calculated from the initial static viscosity values by the following equation:

3 $${\mathrm{Viscosity}} = \frac{{V_s}}{d}$$

where *d* is density of the sample. The density of microgel suspensions and emulsions were assumed to be 0.997 and 0.980 g cm^−3^, respectively, based on that of water (0.997 g cm^−3^) and soybean oils (0.917 g cm^−3^) at 25 °C. All the measurements were performed at 25.0 ± 0.5 °C, and each sample was measured three times.

### Interfacial tension measurements

To precisely evaluate interfacial activity of the prepared polysaccharide microgels, we first conducted soybean oil purification to remove slightly existing surface active polar compounds such as fatty acids and mono- and di-acylglycerols from the oil according to the previous research by Murphy et al.^47^ The interfacial tension between the aqueous microgel suspensions and the purified oil was measured based on the pendant drop technique using a drop tensiometer (Tracker, Teclis Scientific, Tassin la demi lune, Rhône, France). The aqueous microgel suspensions were diluted 10 times with deionized water and filled into a glass call (25 ml). The tip of a rising-type needle attached to a syringe filled with the purified oil was immersed into the aqueous microgel suspension, followed by creation of a rising oil drop into the aqueous phase. The surface area of the created oil drop was controlled to 30 mm^2^ and the interfacial tension were time-dependently calculated based on the density of two phases (0.997 g cm^−^^3^ and 0.917 g cm^−3^ for water and oil, respectively) and the drop shape images captured by a CCD camera. Measurements were carried out at 25 °C for 30 min.

### Determination of surface hydrophobicity

The surface hydrophobicity of microgles was determined using a fluorescence probe, ANS-Na.^48^ The microgel suspensions (0.1–0.5 wt% polysaccharides) were mixed with an aliquot (50 μl) of a 8 mM ANS-Na solution, and the mixture was subsequently incubated in a water bath at 25 °C for 10 min, followed by fluorescence measurements by a spectrofluorometer (FP-8300, JASCO corporation, Tokyo, Japan) with the excitation and emission wave lengths of 390 and 470 nm, respectively. A slope of the biplot of the polysaccharide concentration and arbitrary fluorescence intensity was used as a surface hydrophobicity index.

### Evaluation of physical stability of microgel-stabilized emulsions

#### Observation of creaming behavior

The emulsions were mixed with an aliquot of sodium azide solution to achieve the final concentration of approximately 0.02 wt% and filled into 30 ml of vials immediately after the preparation. The vials were placed on a flat table at 20 °C for 24 h, and the creaming behavior was observed every 30 min. The creaming index was calculated using the following equation:

4 $${\mathrm{Creaming}}\,{\mathrm{index(\% ) = }}\frac{{H_s}}{{H_t}}$$

where *H*_*t*_ and *H*_*s*_ are total height and serum layer height of the emulsions, respectively. The initial creaming rate of the microgel-stabilized emulsions was also determined by linear correlation of the creaming rate plots within the first 2 h. The obtained creaming rate values were compared with theoretical creaming rate calculated by the following equations:^1^

5 $$V_0 = \frac{{D^2\left( {\rho _w - \rho _o} \right)g}}{{{\mathrm{18}}\eta }}$$

where *V*_0_ is the creaming velocity calculated by Stokes' equation, *D* is the oil droplet diameter, *ρ*_w_ and *ρ*_o_ are the density of water and oil (0.997 and 0.917, respectively), and *g* is the acceleration due to gravity. The obtained viscosity values of the microgel suspensions were used as *η* in this study.

#### Evaluation of flocculation and coalescence

The microgel-stabilized emulsions containing 0.02 wt% of sodium azide were stored in an incubator at 25 °C for 8 weeks and subjected to periodical particle size measurements every week. The emulsion was gently mixed by turning the tube upside down 10 times and diluted with an equal volume of water with/without 2 wt% SDS before measurements.^40^

#### Quantification of accelerated oil phase separation

The emulsions (10 ml) were centrifuged at 20,000 × *g* for 30 min at 25 °C. The forcibly destabilized free oils were quantified according to Palanuwech et al.^49^ Briefly, after the centrifugation, bulk oils at the top released from the emulsion systems were mixed with 2 g of additional soybean oils containing 0.002 wt% of Oil Red O, followed by a centrifugation under the same conditions to completely remove contaminants. The absorbance of colored oils at 517 nm was measured. The mass fraction of released free oils, *ϕ*_d_, was calculated using the following equation:^49^

6 $$\phi _d = \frac{{m_o\left( {a - {\mathrm{1}}} \right)}}{{m_e\phi _e}}$$

where *ϕ*_e_ is the mass fractions of oils in the original emulsion, *m*_o_ and *m*_e_ are respective weight of the added Oil Red O solutions and the emulsions, and *a* is absorbance measured at 517 nm, respectively.

#### Confocal laser scanning microscopy

Agar and curdlan were stained by dissolving Nile Blue A and ANS-Na into the microgel suspensions to achieve the final concentration of 0.1 and 0.5 wt%, respectively. Oils were dyed with Nile Red (0.1 wt%) prior to emulsification. The sample was observed without any dilutions using a confocal laser scanning microscope (FV1000, Olympus Corporation, Tokyo, Japan). The excitation and emission wavelengths were 635 and 647 nm for Nile Blue A, 405 and 422 nm for ANS-Na, and 473 and 520 nm for Nile Red, respectively.

#### Statistical analysis

All samples were prepared at least three times. The obtained values were reported as means ± S.D. based on triplicate experiments. Representative images were shown for microscopic and visual observations. All statistical analyses were performed by Microsoft Excel version 2016. The statistical significance among the differently prepared microgels was tested by analysis of variance (*p* = 0.05).

### Data availability

The datasets generated during and/or analyzed during the current study are available from the corresponding author on reasonable request.
